# Interrelation between hypoxic liver injury and Killip classification in ST-segment elevation myocardial infarction patients

**DOI:** 10.3389/fcvm.2024.1396243

**Published:** 2025-01-20

**Authors:** Seong Huan Choi, Ji-Hun Jang, Dae-Young Kim, Young Ju Suh, Yong-Soo Baek, Sung-Hee Shin, Seong-Ill Woo, Dae-Hyeok Kim, Jeonggeun Moon, Jon Suh, WoongChol Kang, Sang-Don Park, Sung Woo Kwon

**Affiliations:** ^1^ Department of Cardiology, Inha University Hospital, Incheon, Republic of Korea; ^2^ Department of Biomedical Sciences, College of Medicine, Inha University, Incheon, Republic of Korea; ^3^ Department of Cardiology, Gil Medical Center, Gachon University, Incheon, Republic of Korea; ^4^ Department of Cardiology, Soon Chun Hyang University Bucheon Hospital, Bucheon, Republic of Korea

**Keywords:** STEMI, HLI, Killip classification, PCI, percutaneous coronary intervention, all-cause mortality

## Abstract

**Introduction:**

Hypoxic liver injury (HLI) and Killip classification are poor prognostic factors in patients with ST-segment elevation myocardial infarction (STEMI). This study investigates the interrelationship between hypoxic liver injury (HLI) and Killip classification.

**Method and results:**

A total of 1,537 STEMI patients who underwent percutaneous coronary intervention (PCI) from 2007 to 2014 at four tertiary hospitals in the Incheon-Bucheon province were enrolled in this study. The patients were divided into four groups based on their Killip classification at presentation in the emergency room (ER). HLI was defined as a ≥2-fold increase in serum aspartate transaminase (AST). The incidence of HLI showed incremental tendency with respect to the Killip classification (19.5%, 19.4%, 34.6%, and 37.8%, respectively; *p* < 0.001). Left ventricular ejection fraction (LVEF) was below 45% in symptomatic, overt heart failure patients (Killip class II, III, and IV). Both initial and peak AST levels increased in accordance with Killip classification along with cardiac biomarkers. In-hospital mortality was directly related to Killip classification (2.3%, 7.3%, 16.3%, 29.2%) with statistical significance. Univariate and multivariate Cox regression analysis showed that the presence of HLI and combined Killip classification III and IV were poor prognostic factors, even after adjusting for conventional clinical risk factors. Receiver operating characteristic (ROC) analysis showed that combination of HLI and Killip classification was the most sensitive predictor of mortality (AUC 0.832, 95% CI 0.78–0.882). Kaplan–Meier curve showed that patients with HLI and Killip class (III and IV) had the lowest event-free survival regarding in-hospital mortality and major cardiovascular and cerebrovascular events.

**Conclusions:**

The presence of HLI and Killip classification were directly related to worse prognosis in STEMI patients. Early recognition of HLI and accurate assessment of Killip classification is warranted for effective management of STEMI.

## Introduction

Consequential results from decades of evolutionary innovation regarding percutaneous coronary intervention (PCI) have enabled ST-segment elevation myocardial infarction (STEMI) patients with improved overall survival (1–3). Growing emphasis on timely PCI, advancement of imaging devices helping to optimize PCI procedure, and introduction of highly potent P2Y12 inhibitors to minimize acute stent thrombosis have all contributed to the reduction of procedure-associated and mechanical complications of STEMI (4–6). However, despite these efforts to maximize survival and minimize complications, the overall mortality is still relatively high in STEMI patients (7, 8). Clinical risk scoring systems stemming back from the 1980s to as early as 2020 have paved ways to predict and enhance the survival of STEMI patients. Thrombolysis in myocardial infarction (TIMI) score was first derived to predict survival of STEMI patients utilizing relevant clinical risk factors, and the global registry of acute coronary event (GRACE) score, which stratifies risk scores for early intervention, was devised to further extend the concept of early prediction of clinical prognosis (9–11). Nevertheless, even with unrelenting enthusiasm from cardiovascular researchers, the scoring system devised to evaluate and predict clinical outcomes seemed to be rather vague and ambiguous for physicians in clinical practice. STEMI is a clinical phenomenon that requires urgent, decisive, and comprehensive medical attention (12). The most common clinical manifestation companied by STEMI is heart failure. The inability of systolic cardiac function due to damaged myocardium causes insufficient systemic circulation causing diverse symptoms ranging from mild dyspnea to cardiogenic shock (13). Another clinical phenomenon that ensues specifically from circulatory dysfunction is hypoxic liver injury (HLI). Inadequate supply of oxygen to the liver caused by depressed heart function results in hepatic ischemia (14, 15). Each of the aforementioned clinical phenomena has apprehensive features regarding prognosis. Escalation of heart failure, most commonly described as Killip classification, is a well-known parameter for mortality in STEMI. HLI is another caveat for STEMI patients with matters pertaining to mortality. Despite the clinical importance these two distinctive phenomena possess in STEMI patients, the clinical impact with which they have upon each other has not been fully dealt with. Therefore, we aim to investigate the interrelationship between HLI and Killip classification in STEMI patients.

## Method

### Study protocol and data acquisition

Our current study was a retrospective observational study approved by the Review Board of the Inha University Hospital, Inha University College of Medicine, and each patient's written consent form was waived by the review board. The clinical data obtained from the registry were accessible for research purposes on 15 December 2015. The authors had no access to information that could possibly identify participating individuals. Four tertiary hospitals (i.e., Inha University Hospital, Gacheon University Gil Hospital, Sejong General Hospital, and Soon Chun Hyang University Bucheon Hospital) in the Incheon-Bucheon province comprised a registry of STEMI patients who had received primary percutaneous coronary intervention [**IN**cheon-Bucheon cohor**T** of patients und**ER**went primary PCI for acute **ST El**evation myocardial infarction (INTERSTELLAR)]. STEMI patients who had undergone primary PCI from 2007 to 2014 were enrolled. Patients with a prior history of any form of hepatitis were excluded including chronic hepatitis, viral hepatitis, alcoholic liver disease, or toxic hepatitis. The coronary intervention was performed in accordance with the current guidelines for coronary revascularization (16). Pharmacological treatment and mechanical support related to primary PCI were left to the operator's discretion. Patient Killip classification and the presence of hypoxic liver injury (HLI) were assessed at the time of the initial emergency room (ER) visit.

### Definition of variables and measurements

Systolic blood pressure (SBP) above 140 mmHg, diastolic blood pressure above 90 mmHg, and prior use of antihypertensive medication were defined as having hypertension (HTN). The definition of diabetes mellitus (DM) was as follows: (1) prior use of hypoglycemic agents or insulin, (2) fasting plasma glucose above 126 mg/dl or glycosylated hemoglobin (HbA1c) above 6.5%, and (3) previously diagnosed but untreated hyperglycemia. Dyslipidemia was defined by the following: total cholesterol ≥240 mg/dl, LDL cholesterol ≥130 mg/dl, HDL cholesterol <40 mg/dl, triglycerides ≥200 mg/dl, and prior use of lipid-lowering agents. A patient who was currently smoking or had smoked until 1 month prior to primary PCI was considered a smoker. Diagnosis of STEMI was achieved when an electrocardiogram showed an ST elevation of >1 mm in at least two consecutive leads or new-onset left bundle branch block, twofold elevation of serum levels of troponin I or creatine kinase-MB above the upper normal limit, and typical anginal chest pain lasting for more than 30 min. Coronary artery disease (CAD) was defined as luminal narrowing of >50% in any epicardial coronary artery. (17). HLI was defined as a ≥2-fold increase in serum aspartate transaminase (AST) above the upper normal limit at admission (18).

### Primary and secondary clinical endpoints

Our primary endpoint was all-cause in-hospital mortality with respect to the patient's Killip classification combined with the presence of HLI at the ER. Our secondary endpoint was major cardiovascular and cerebrovascular events (MACCE) commensurate with Killip classification and the presence of HLI. MACCE were defined as incidence or episodes of death, myocardial infarction (MI), target vessel revascularization (TVR)/target lesion revascularization (TLR), coronary artery bypass grafting (CABG), ischemic stroke, and hemorrhagic stroke during follow-up period. Follow-up clinical data were collected through either electronic medical record review or standardized telephone interviews.

### Data analysis and statistical methods

Continuous data were presented as mean value ± standard deviation. Categorical data were presented as percentage or absolute number. Analyses of continuous data were performed using the analysis of variance (ANOVA) test, and analyses of categorical data were performed using the chi-square test to assess differences among the four groups. Receiver operating characteristic (ROC) analysis was applied to evaluate the predictive value of HLI at ER combined with Killip classification on in-hospital mortality (19). Multivariate and univariate Cox regression analyses were performed to determine the risk factors associated with in-hospital mortality and MACCE which included clinical characteristics, laboratory findings, Killip classification, LVEF, and HLI. Hazard ratios (HR) were calculated as an estimate of the risk associated with a particular variable with 95% confidence intervals (CI). All analyses were performed using SPSS version 19.0 (SPSS, Chicago, IL, USA) and SAS version 9.3 (SAS Institute, Cary, NC, USA). A *p*-value of <0.05 was considered statistically significant.

## Results

### Baseline characteristics of the study population

A total of 1,516 patients were enrolled. The number of patients in Killip classes I, II, III, and IV was 1,185, 108, 104, and 119, respectively. The mean age for each group was as follows: Killip class I, 59.34 ± 12.88; Killip class II, 63.35 ± 13.95; Killip class III, 66.74 ± 13.64; and Killip class IV, 63.39 ± 12.85 (*p* < 0.001). Male gender was predominant in all four groups (80.6%, 76.1%, 68.3%, 79.2%; *p* = 0.023). The prevalence of DM showed incremental tendency in accordance with the groups’ Killip classification (23.9%, 29.4%, 41.3%, 43.3%; <0.001). Initial liver enzyme (AST/ALT) level increased gradually as Killip classification deteriorated; Killip class I, 61.60 ± 91.94; Killip II, 70.81 ± 95.25; Killip III, 87.74 ± 112.78; and Killip IV, 139.82 ± 345.53 (*p* < 0.001). Initial creatine kinase (CK) and CK-MB level did not show any association with Killip classification (initial CK; Killip I, 493.20 ± 1,123.67; Killip II, 471.50 ± 809.76; Killip III, 551.81 ± 846.28; Killip IV, 466.07 ± 736.85; *p* = 0.93) (initial CK-MB; Killip I, 44.43 ± 170.25; Killip II, 31.25 ± 65.86; Killip III, 53.73 ± 105.59; Killip IV, 80.06 ± 387.61; *p* = 0.19). However, peak CK and CK-MB levels were directly related to Killip classification (peak CK: Killip I, 1,759.91 ± 2,357.527; Killip II, 1,921.17 ± 3,237.93; Killip III, 2,194.17 ± 4,126.27; Killip IV, 3,135.89 ± 4,555.88; *p* < 0.001) (peak CK-MB: Killip I, 199.92 ± 208.87; Killip II, 197.07 ± 201.39; Killip III, 248.65 ± 299.10; Killip IV, 297.80 ± 317.48; *p* < 0.001). Killip class I patients showed nearly preserved left ventricular ejection fraction (LVEF), but for Killip class (II, III, IV), the LVEF was mildly reduced (Killip I, 49.75 ± 11.12; Killip II, 43.55 ± 12.84; Killip III, 41.24 ± 12.74; Killip IV, 43.52 ± 15.99; *p* < 0.001). Multivessel disease, left main (LM) artery disease involvement, and the use of intra-aortic balloon pump (IABP), all of which translate to disease severity, were directly associated with Killip classification. In-hospital mortality and the presence of hypoxic liver injury (HLI) increased accordingly with respect to Killip classification [in-hospital mortality; Killip class I, 27 (2.3%); Killip II, 8 (7.3%); Killip III, 17 (16.3%); Killip IV, 35 (29.2%); *p* < 0.001] [HLI; Killip I, 231(19.5%); Killip II, 21 (19.4%); 36 (34.6%); Killip IV, 45 (37.8%); *p* < 0.001] (Table 1).

**Table 1:** Baseline characteristics.

Baseline characteristics	Killip class I (1,185)	Killip class II (108)	Killip class III (104)	Killip class IV (119)	*p*-value
Age (years)	59.34 ± 12.88	63.35 ± 13.95	66.74 ± 13.64	63.39 ± 12.85	<0.001
Male gender (%)	957 (80.6%)	83 (76.1%)	71 (68.3%)	95 (79.2%)	0.023
Diabetes (%)	284 (23.9%)	32 (29.4%)	43 (41.3%)	52 (43.3%)	<0.001
HTN (%)	553 (46.5%)	48 (44.0%)	67 (64.4%)	69 (57.5%)	0.001
Dyslipidemia (%)	232 (19.5%)	18 (16.5%)	27 (26.0%)	25 (20.8%)	0.34
Current smoking (%)	803 (67.6%)	62 (56.9%)	48 (46.2%)	69 (57.5%)	<0.001
SBP (mmHg)	128.19 ± 26.513	123.45 ± 27.476	124.14 ± 30.05	90.72 ± 30.07	<.0001
DBP (mmHg)	78.57 ± 16.65	75.27 ± 16.94	75.58 ± 19.34	55.09 ± 21.86	<0.001
Initial AST (mg/dl)	61.60 ± 91.94	70.81 ± 95.25	87.74 ± 112.78	139.82 ± 345.53	<0.001
Initial ALT (mg/dl)	34.23 ± 26.91	37.49 ± 49.25	51.04 ± 54.51	79.48 ± 161.77	<0.001
Peak AST (mg/dl)	217.46 ± 318.07	244.83 ± 213.81	434.08 ± 1,354.23	833.11 ± 2,304.79	<0.001
Peak ALT (mg/dl)	73.446 ± 232.46	77.64 ± 85.46	181.86 ± 671.23	346.42 ± 1,218.37	<0.001
Initial CK (U/L)	493.20 ± 1,123.67	471.50 ± 809.76	551.81 ± 846.28	466.07 ± 736.85	0.93
Initial CK-MB (ug/ml)	44.43 ± 170.25	31.25 ± 65.86	53.73 ± 105.59	80.06 ± 387.61	0.19
Initial Tnl (ng/ml)	8.91 ± 29.69	15.45 ± 54.96	13.21 ± 54.29	29.65 ± 146.37	0.003
Peak CK (U/L)	1,759.91 ± 2,357.527	1,921.17 ± 3,237.93	2,194.17 ± 4,126.27	3,135.89 ± 4,555.88	<0.001
Peak CK-MB (ug/ml)	199.92 ± 208.87	197.07 ± 201.39	248.65 ± 299.10	297.80 ± 317.48	<0.001
Peak Tnl (ng/ml)	53.717 ± 96.11	40.93 ± 116.51	75.51 ± 93.82	48.65 ± 80.62	0.31
LVEF (%)	49.75 ± 11.12	43.55 ± 12.84	41.24 ± 12.74	43.52 ± 15.99	<0.001
CAD extent					
One-vessel disease (%)	486 (40.9%)	40 (36.7%)	30 (30.9%)	42 (35.6%)	0.166
Two-vessel disease (%)	394 (33.2%)	40 (36.7%)	30 (30.9%)	40 (33.9%)	0.84
Three-vessel disease (%)	307 (25.8%)	29 (26.6%)	37 (38.1%)	36 (30.5%)	0.054
Multivessel disease (%)	657 (55.7%)	66 (61.7%)	63 (65.6%)	77 (65.3%)	0.049
Multivessel PCI (%)	311 (48.1%)	22 (33%)	35 (55%)	41 (57.7%)	0.634
Multivessel staged PCI (%)	100 (32.1%)	8 (36.3%)	12 (34.2%)	11 (26.8%)	0.692
Infarct-related artery					
LAD (%)	601 (50.6%)	64 (58.7%)	61 (62.9%)	42 (35.6%)	<0.001
LCX (%)	133 (11.2%)	6 (5.5%)	9 (9.3%)	11 (9.3%)	0.281
RCA (%)	454 (38.2%)	39 (35.8%)	27 (27.8%)	65 (55.1%)	<0.001
LM (%)	14 (1.2%)	3 (1.9%)	5 (5.2%)	8 (6.8%)	<0.001
IABP (%)	21 (1.8%)	4 (3.8%)	11 (11.5%)	20 (17.2%)	<0.001
STB (min)	637.43 ± 1,953.42	576.27 ± 1,316.04	518.53 ± 989.38	471.97 ± 947.84	0.845
In-hospital mortality (%)	27 (2.3%)	8 (7.3%)	17 (16.3%)	35 (29.2%)	<0.001
Hypoxic liver injury (%)	231(19.5%)	21(19.4%)	36(34.6%)	45(37.8%)	<0.001

HTN, hypertension; SBP, systolic blood pressure; DBP, diastolic blood pressure; AST, aspartate transaminase; ALT, alanine transaminase; CK, creatinine kinase; TnI, troponin I; LVEF, left ventricular ejection fraction; CAD, coronary artery disease; PCI, percutaneous coronary intervention; LAD, left anterior descending artery; LCX, left circumflex artery; RCA, right coronary artery; LM, left main; IABP, intra-aortic balloon pump; STB, symptom to balloon time.

### Primary and secondary clinical outcomes

**Figure 1:**
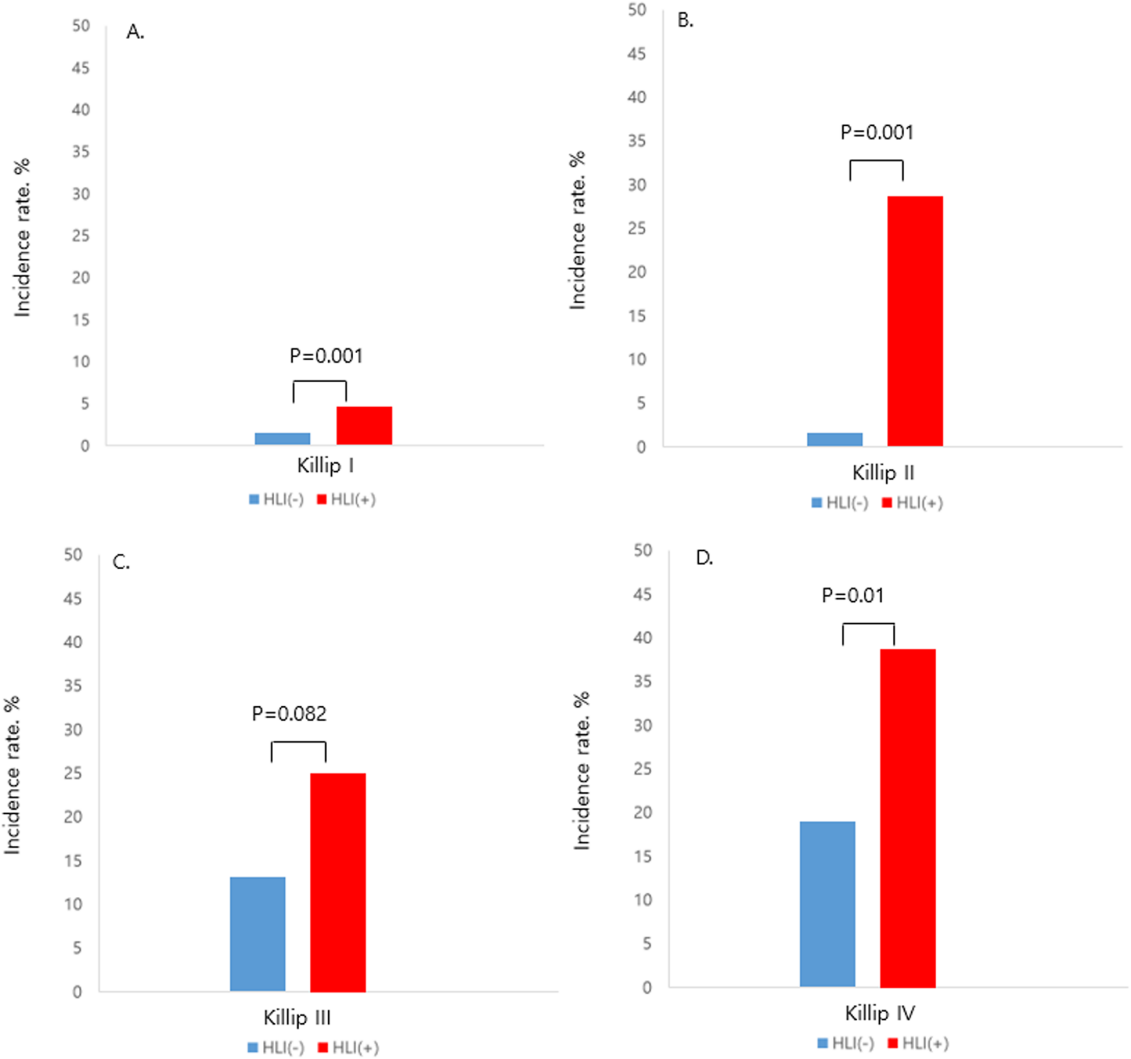
Incidence of in-hospital mortality according to the presence of HLI in each Killip class, HLI, hypoxic liver injury. **(A)** Killip class I. **(B)** Killip class II. **(C)** Killip class III. **(D)** Killip class IV.

**Figure 2:**
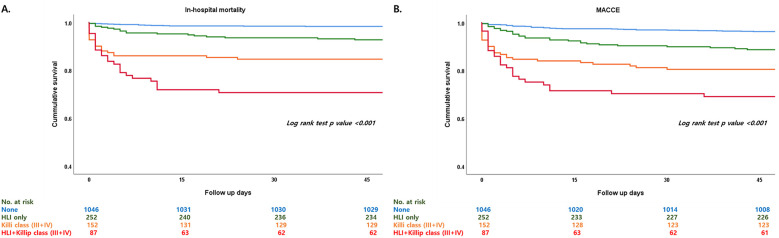
Kaplan–Meier curve comparing HLI, Killip class III and IV, and combination of HLI and Killip class III and IV regarding in-hospital mortality and MACCE. HLI, hypoxic liver injury; MACCE, major adverse cardiovascular and cerebrovascular event. **(A)** In-hospital mortality, **(B)** MACCE.

**Figure 3:**
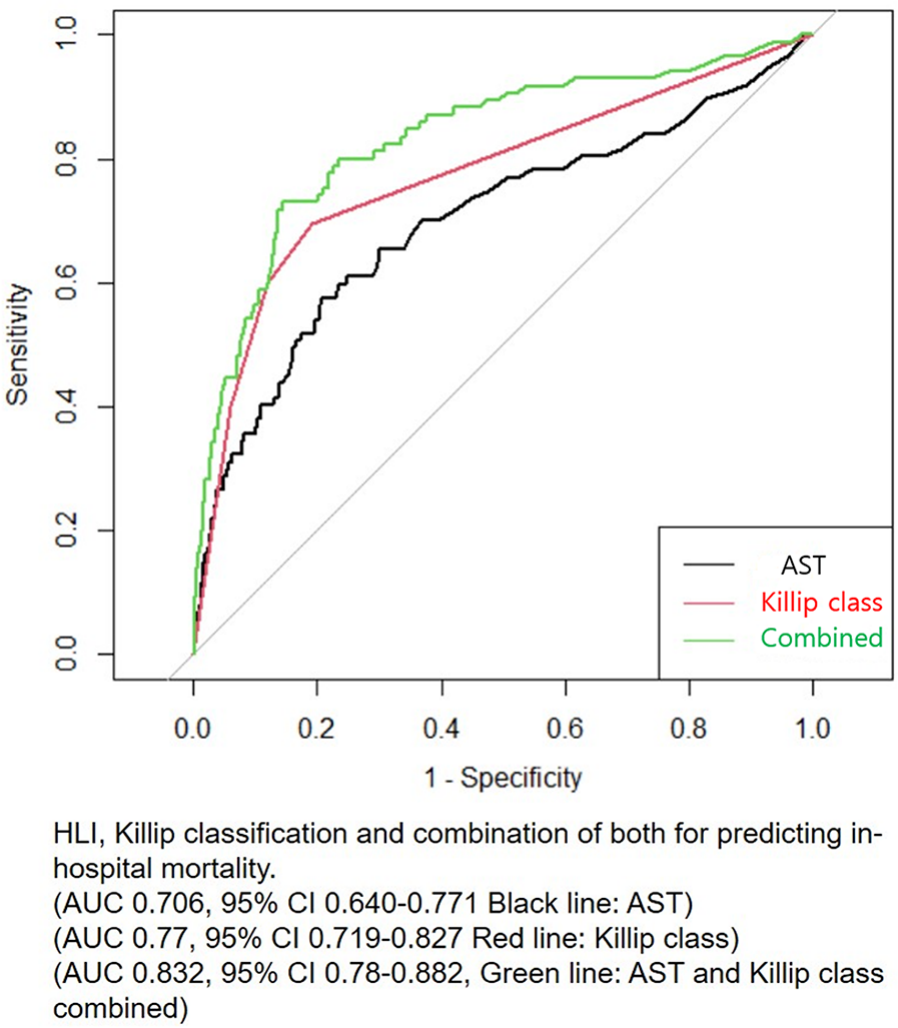
Receiver operating characteristic (ROC) analysis for predicting in-hospital mortality. AST, aspartate transaminase; HLI, hypoxic liver injury.

Patients who had HLI showed higher in-hospital mortality in every Killip classification respectfully (Killip I, 1.5% vs. 5.2%; Killip II, 2.3% vs. 28.6%; Killip III, 11.8% vs. 25%; Killip IV, 20.3% vs. 42.2%) (Figure 1). Multivariate and univariate Cox regression model for in-hospital mortality (Killip class III and IV: HR = 2.671, 95% CI 1.376–5.185, *p* = 0.004) (HLI: HR = 3.989, 95% CI 2.058–7.730, *p* < 0.001) and MACCE (Killip class III and IV: HR = 2.015, 95% CI 1.380–2.943, *p* < 0.001) (HLI: HR = 1.681, 95% CI 1.200–2.356, *p* = 0.003) showed that even after adjusting for clinical and classical risk factors regarding mortality and MACCE, Killip class III and IV (overt heart failure and cardiogenic shock) and HLI were poor prognostic factors (Table 2). Cox regression model comparing HLI, Killip class III and IV, and combination of HLI and Killip class III and IV showed that the combination of the two factors had the worst event-free survival regarding in-hospital mortality (HR = 9.445, 95% CI 3.857–23.128, *p* < 0.001) and MACCE (HR = 3.512, 95% CI 2.040–6.046, *p* < 0.001) (Table 3). Kaplan–Meier curve for event-free probability for in-hospital mortality and MACCE showed that patients who were in Killip class III and IV conditions combined with the presence of HLI had the lowest event-free survival (Figure 2). ROC analysis to evaluate the predictive value of Killip classification, initial AST, and the two factors combined for in-hospital mortality showed that the combination of the two factors had a synergistic influence upon each factor (AUC 0.832, 95% CI 0.78–0.882) (Figure 3).

**Table 2:** Univariate and multivariate Cox regression model for in-hospital mortality and MACCE.

In-hospital mortality						
Variable	Univariate			Multivariate		
	HR	95% CI	*p*-value	HR	95% CI	*p*-value
Age	1.055	1.037–1.073	<0.001	1.046	1.020–1.073	<0.001
Male	0.528	0.339–0.822	0.005			
DM	2.398	1.580–3.638	<0.001			
HTN	1.75	1.141–2.685	0.01			
Dyslipidemia	0.711	0.395–1.280	0.256			
LVEF	0.906	0.888–0.924	<0.001	0.933	0.908–0.959	<0.001
Serum creatinine	1.15	1.055–1.255	0.002			
Symptom to balloon time	1.053	0.943–1.177	0.357			
Door to balloon time	1.32	0.797–2.187	0.281			
Multivessel disease	2.653	1.594–4.415	<0.001			
Left main artery disease	4.897	2.714–8.836	<0.001			
Killip class III and IV	9.518	6.218–14.571	<0.001	2.671	1.376–5.185	0.004
Hypoxic liver injury	4.308	2.826–6.588	<0.001	3.989	2.058–7.730	<0.001
MACCE						
Age	1.036	1.026–1.046	<0.001	1.029	1.016–1.042	<0.001
Male	0.798	0.597–1.068	0.129			
DM	1.777	1.372–2.302	<0.001			
HTN	1.445	1.125–1.857	0.004			
Dyslipidemia	1.028	0.750–1.409	0.866			
LVEF	0.949	0.939–0.960	<0.001	0.959	0.946–0.972	<0.001
Serum creatinine	1.123	1.058–1.193	<0.001	1.117	1.026–1.215	0.011
Symptom to balloon time	1.04	0.961–1.125	0.333			
Door to balloon time	1.116	0.770–1.619	0.562			
Multivessel disease	1.719	1.309–2.258	<0.001			
Left main artery disease	3.157	2.036–4.896	<0.001	1.972	1.118–3.478	0.019
Killip class III and IV	3.695	2.841–4.806	<0.001	2.015	1.380–2.943	<0.001
Hypoxic liver injury	1.93	1.484–2.511	<0.001	1.681	1.200–2.356	0.003

HR, hazard ratio; CI, confidence interval; HLI, hypoxic liver injury; MACCE, major cardiovascular and cerebrovascular event; DM, diabetes mellitus; HTN, hypertension; LVEF, left ventricular ejection fraction.

**Table 3:** Univariate and multivariate Cox regression model comparing HLI, Killip class III and IV, and combination of HLI and Killip III and IV for in-hospital mortality and MACCE.

In-hospital mortality						
HLI and Killip classification	Univariate			Multivariate		
	HR	95% CI	*p*-value	HR	95% CI	*p*-value
Reference						
HLI	4.227	2.199–8.123	<0.001	3.196	1.409–7.252	0.005
Killip class III and IV	10.299	5.589–18.981	<0.001	1.98	0.672–5.832	0.215
HLI and Killip class III and IV	22.907	12.713–41.275	<0.001	9.445	3.857–23.128	<0.001
MACCE						
Reference						
HLI	1.589	1.130–2.235	0.008	1.553	1.045–2.3,096	0.029
Killip class III and IV	3.284	2.328–4.631	<0.001	1.904	1.177–3.078	0.009
HLI and Killip class III and IV	5.568	3.873–8.003	<0.001	3.512	2.040–6.046	<0.001

HR, hazard ratio; CI, confidence interval; HLI, hypoxic liver injury; MACCE, major adverse cardiovascular and cerebrovascular event.

^a^ Adjusted variables are age, male gender, diabetes mellitus, hypertension, dyslipidemia, left ventricular ejection fraction, symptom to balloon time, door to balloon time, multivessel disease, and left main artery disease.

## Discussion

Our current study collected clinical data from a multicenter consortium (INTERSTELLAR) designed specifically to investigate clinical features of STEMI patients who had undergone PCI. Among the study population, patients were initially divided according to their Killip classification, and the presence of HLI was determined utilizing guidelines from previous study protocols (20–22). In patients with HLI at index visits from the emergency room (ER) combined with Killip classification III and IV, the risk of in-hospital mortality and MACCE were much higher. Even with statistical adjustments with clinical and classical risk factors, the combination of the presence of HLI and Killip classification III and IV showed a detrimental synergistic tendency regarding in-hospital mortality and MACCE.

With growing technological evolution and clinical implementation of new drug-eluting stents (DES) embedding state-of-the-art biotechnology and highly potent anti-platelet agents to maximize stent integrity, these advancements in the medical field have shed light on the improvement of mortality and symptom-free period (23). Even with these improvements, many researchers have investigated whether there were any amenable aspects that could benefit STEMI patients. Historically, the most efficient way of discerning risk factors or prognostic factors affecting the patient in a real-world clinic is by utilizing an intuitive and sophisticated risk-assessing system. TIMI risk scoring system was devised to stratify STEMI patients with clinically associated risk factors which ultimately translated to short-term and long-term prognosis (24). Further research regarding prognostic factors related to mortality paved the way for newer and more sophisticated scoring systems to predict clinical outcomes. The Global Registry of Acute Coronary Events (GRACE) scoring system was able to show that by combining clinical biomarkers specific to ACS, risk stratification was more comprehensive, and prediction of disease severity and outcome became more feasible (25). Killip classification, one of the most common classifications used to stratify ACS patients with heart failure, is a simple but very efficient risk assessment system. It is known that patients with high Killip class followed by left ventricular dysfunction have the highest short-term and long-term mortality (13, 26). A higher Killip class was associated with higher gastrointestinal bleeding risk in ACS patients, and even in patients without obstructive coronary artery disease such as myocardial infarction with the non-obstructive coronary artery (MINOCA), Killip classification showed an incremental tendency toward worse clinical outcomes (27, 28).

Recently, the concept of organ dysfunction as a surrogate parameter for clinical outcome and the clinical impact it can have with early detection of such organ dysfunction in STEMI patients drew the attention of many researchers (29–31). Numerous studies have elaborated on the clinical impact and benefit of combining risk scoring systems with biomarkers and organ dysfunction in STEMI patients. However, incorporating Killip classification, one of the most clinically driven stratifying tools, with organ dysfunction is relatively sparse. In our current study, patients with high Killip classification were at higher risk of in-hospital mortality. The use of an intra-aortic balloon pump (IABP), involvement of the left main (LM) artery, and multivessel disease, which translates to disease severity, increased as Killip class deteriorated. The presence of HLI also showed incremental tendency with respect to Killip classification, and in-hospital mortality was higher in patients with HLI in all Killip classification categories. The Cox regression model adjusting for clinical risk factors showed that both HLI and Killip class III and IV were independent risk factors regarding in-hospital mortality and MACCE. A combination of the two clinical phenotypes showed that they had an augmentative influence on each phenomenon which was demonstrated by the Kaplan–Meier survival curve and ROC analysis.

Past risk scoring systems relied on predisposing factors or consequential assessment after the initial ACS event. However, Killip classification stratifies patients with their presenting concomitant phenotype at index ER visit, and the definition of HLI is determined by baseline lab results taken at index visit of a medical institution. Therefore, these clinical parameters are evident at an early phase of medical contact for treatment which provides useful clinical information to the healthcare provider and acknowledges that the combination of the presence of HLI and Killip class III and IV are early signs of multiorgan dysfunction in the attending physician can be more attentive whilst treating STEMI patients.

Our study has some limitations. Firstly, our study is retrospective; therefore, we cannot suggest any medical recommendations to improve clinical outcomes regarding HLI and Killip classification. Our study design does not allow us to assert any clinical argument regarding the treatment of the combined HLI and Killip class III and IV patients but only assumes that they were at a higher risk of primary and secondary outcomes. Therefore, further studies postulating the effect of early detection of HLI and Killip classification with active medical intervention are warranted. Secondly, we were not able to acquire data regarding the medication applied to the ACS patients upon their arrival to the hospital for admission. Our study population consists of ACS patients therefore adequate medical attention from the first medical contact to the arrival to the emergency room is crucial for overall survival. Thirdly, despite being a multicenter study, it has a relatively small population in general compared to other registries and is comprised only of Southeast Asians, which would ultimately be insufficient to portray the entire STEMI patients.

In conclusion, HLI and Killip class III and IV were independent risk factors for in-hospital mortality and MACCE in STEMI patients. The combination of the two distinctive phenotypes showed detrimental synergistic results regarding in-hospital mortality and MACCE. Therefore, early detection of HLI and accurate assessment of Killip classification is warranted in treating STEMI patients.

## Data Availability

The raw data supporting the conclusions of this article will be made available by the authors, without undue reservation.
